# Effect of oral acetyl L-carnitine arginate on resting and postprandial blood biomarkers in pre-diabetics

**DOI:** 10.1186/1743-7075-6-25

**Published:** 2009-06-02

**Authors:** Richard J Bloomer, Kelsey H Fisher-Wellman, Patrick S Tucker

**Affiliations:** 1 Cardiorespiratory/Metabolic Laboratory, The University of Memphis, Memphis, Tennessee 38152, USA

## Abstract

**Background:**

Resting and postprandial oxidative stress is elevated in those with metabolic disorders such as diabetes. Antioxidant supplementation may attenuate the rise in oxidative stress following feeding. Therefore we sought to determine the effects of acetyl L-carnitine arginate (ALCA) on resting and postprandial biomarkers of glucose and lipid metabolism, as well as oxidative stress.

**Methods:**

Twenty-nine pre-diabetic men and women were randomly assigned to either 3 g·day^-1 ^of ALCA (n = 14; 31 ± 3 yrs) or placebo (n = 15; 35 ± 3 yrs) in a double-blind design, to consume for eight weeks. Fasting blood samples were taken from subjects both pre and post intervention. After each fasting sample was obtained, subjects consumed a high fat, high carbohydrate meal and additional blood samples were taken at 1, 2, 4, and 6 hours post meal. Samples were analyzed for a variety of metabolic variables (e.g., glucose, HbA1c, lipid panel, C-reactive protein, nitrate/nitrite, and several markers of oxidative stress). Area under the curve (AUC) was calculated for each variable measured post meal, both pre and post intervention.

**Results:**

ALCA, but not placebo, resulted in an increase in nitrate/nitrite (25.4 ± 1.9 to 30.1 ± 2.8 μmol·L^-1^) from pre to post intervention, with post intervention values greater compared to placebo (p = 0.01). No other changes of statistical significance were noted (p > 0.05), although ALCA resulted in slight improvements in glucose (109 ± 5 to 103 ± 5 mg·dL^-1^), HbA1c (6.6 ± 1.1 to 6.2 ± 1.2%), and HOMA-IR (3.3 ± 1.3 to 2.9 ± 1.2). AUC postprandial data were not statistically different between ALCA and placebo for any variable (p > 0.05). However, nitrate/nitrite demonstrated a moderate effect size (*r *= 0.35) for increase from pre (139.50 ± 18.35 μmol·L^-1^·6 hr^-1^) to post (172.40 ± 21.75 μmol·L^-1^·6 hr^-1^) intervention with ALCA, and the magnitude of decrease following feeding was not as pronounced as with placebo.

**Conclusion:**

Supplementation with ALCA results in an increase in resting nitrate/nitrite in pre-diabetics, without any statistically significant change in other metabolic or oxidative stress variables measured at rest or post meal.

## Background

More than 70 million individuals within the United States alone currently live with cardiovascular disease [1], while nearly 21 million people have diabetes and 54 million are diagnosed with pre-diabetes [2]. Oxidative stress is suggested as one unifying link between cellular dysfunction and the onset and progression of both cardiovascular [3] and metabolic [4] disorders. Although a basal level of radical production is important for proper physiological function [5], overexpression of radical species, either via increased production or decreased antioxidant defense, can result in oxidative damage to lipids, proteins, and nucleic acids, all of which may contribute to the pathophysiology of disease [6]. In particular, oxidative damage to the vascular endothelium is well described [7] and is associated with a poor cardiovascular prognosis [8].

Meals high in fat and carbohydrate that lead to hypertriglyceridemia or hyperglycemia have been shown to promote an exacerbation in radical production [9] and have been associated with increased risk for atherosclerosis and related disorders [10]. Thus, postprandial oxidative stress (and associated measures of triglycerides and blood glucose) appears to provide more important information related to cardiovascular and metabolic disease development and progression [3,11].

Individuals with impaired glucose metabolism are most susceptible to postprandial oxidative stress, as they typically experience prolonged periods of hyperglycemia [12-14] and hypertriglyceridemia [15,16] post feeding. Elevations in blood glucose and triglycerides are directly associated with superoxide anion production [17], a potent and harmful radical species [18].

Antioxidant intake has been used in several studies in an attempt to attenuate the rise in postprandial oxidative stress. This body of work has included the study of foods such as olive oil [19], almonds [20] and red wine [21], as well as nutritional supplements containing vitamins and minerals [22-24]. One antioxidant nutrient with a significant body of literature in support of its antioxidant effects is carnitine [25,26] which is associated with a reduction in xanthine oxidase activity [27] and known to possess free-radical scavenging activity [28].

A recent report by Volek and colleagues [29] demonstrated the potential benefits of L-carnitine, L-tartrate supplementation on endothelial cell function, assessed via flow mediated dilation (FMD). The authors suggest a potential role of nitric oxide in promoting these findings, as nitric oxide is a signaling molecule known to facilitate vasodilatation by acting on vascular smooth muscle cells [30]. This appears partly supported by preliminary studies involving carnitine supplementation and measurement of nitric oxide in a fasted state [31-33]. However, to our knowledge, no study has investigated the potential benefits of dietary carnitine either alone or in conjunction with L-arginine (the precursor to nitric oxide biosynthesis [34]) on circulating nitric oxide during the postprandial state, and only that of Volek et al. [29] has included a measure of postprandial oxidative stress (malondialdehyde). Because nitric oxide typically decreases in response to an increase in postprandial oxidative stress [22,35], likely mediated by the increase in superoxide production and associated endothelial dysfunction [36], maintenance of nitric oxide via carnitine ingestion could be viewed as favorable in regards to vascular function. This may be of particular importance for those with metabolic dysfunction.

Therefore, the purpose of this study was to determine the effects of supplementation with a novel L-arginine containing form of acetyl L-carnitine supplementation (ArginoCarn^®^) on postprandial oxidative stress and associated metabolic biomarkers in a sample of pre-diabetic men and women. We hypothesized that 1) oxidative stress biomarkers would be increased, and nitric oxide decreased, following ingestion of a high fat, high carbohydrate test meal, and 2) subjects assigned to the arginine/carnitine supplement for eight weeks would experience attenuation in postprandial oxidative stress and nitric oxide changes.

## Methods

### Subjects

Forty men and women between the ages of 20 and 55 years were enrolled in this study. Subjects were non-smokers (and not routinely exposed to passive smoke), pre-diabetic (fasting blood glucose: 100–125 mg·dL^-1^), normolipidemic (fasting triglycerides < 200 mg·dL^-1^), and free of cardiovascular, gastrointestinal, or metabolic disorders. Subjects were not currently pregnant, not using antioxidant supplements or drugs during the study period, and not using any type of blood glucose or lipid regulating medication or nutritional supplements. Subjects were required to have a resting blood pressure <160/100 mmHg and a body mass index of 25 kg·m^-2 ^– 40 kg·m^-2^.

### Baseline Assessment

During the first laboratory visit subjects completed health and physical activity questionnaires. Subjects' height, weight, circumference measurements, body mass index, body composition (seven-site testing method [37]) using the Siri equation [38], and resting heart rate and blood pressure (following a 10-minute rest period) were recorded. The same measurements were obtained following the eight week intervention. Methods were approved by the university committee for human subject research, in accordance with the Helsinki Declaration, and all subjects provided both verbal and written consent.

### Treatment

Following the conclusion of the above baseline tests, subjects were provided with study instructions and were randomly assigned in double-blind manner to one of following conditions: Acetyl L-carnitine arginate (ALCA; ArginoCarn^®^, Sigma-tau HealthScience S.p.A, Rome, Italy; n = 20) or Placebo (cellulose; n = 20). The dosage of ALCA was 3 g·day^-1 ^and was taken in two divided doses with meals (morning and evening – 3 capsules at each time). The composition of 3 grams of ALCA provided approximately 1350 mg of acetyl L-carnitine and 1200 mg of arginine. Arginine is a precursor to nitric oxide biosynthesis [34] and has been associated with positive effects related to vasodilatation when used in high dosages (e.g., 15 grams) via either intravenous infusion [39,40] or oral intake [41]. Capsules were identical in appearance and provided to subjects in unlabeled bottles every two weeks, for the duration of the eight week intervention. Capsule counts upon bottle return determined compliance to treatment.

### Meal Testing (pre-intervention and post-intervention)

All subjects consumed two identical tests meals; one before and one following the eight week intervention. Subjects reported to the lab in the morning following a 10-hour overnight fast and a pre-meal blood sample was collected. Subjects then consumed a high-fat, high carbohydrate milkshake, made with whole milk, ice cream, and whipping cream. The caloric load of the test meal was based on subjects' body mass and was equivalent to 1.2 grams of both fat and carbohydrate, and 0.25 grams of protein per kilogram. The test meal provided approximately 17 calories per kilogram of body weight. Blood samples were collected from subjects at 1, 2, 4, and 6 hours following meal intake. Subjects remained in the lab during the observation period and remained inactive. No additional meals or calorie containing beverages were allowed, although the intake of plain water was allowed ad libitum. These exact procedures, including the test meal, have been used in six recent investigations in our lab without incident [42-47]

### Blood Sampling

Venous blood samples (~10 mL per draw) were taken from subjects' forearm via needle and collection tube. These blood samples were collected pre-meal (0 hour), and at 1, 2, 4, and 6 hour postprandial. The plasma/serum was immediately processed and stored at -80°C until analyzed. Blood samples collected at all time points were assayed for malondialdehyde, xanthine oxidase activity, hydrogen peroxide, triglycerides, glucose, trolox equivalent antioxidant capacity (TEAC), and nitrate/nitrite (a surrogate marker of nitric oxide). Blood samples collected at the pre-meal time point only were assayed for insulin, HbA1c, total and HDL cholesterol, and C-reactive protein. Assays were performed on the first thaw. The procedures outlined above for meal testing and blood collection were used both pre- and post-intervention.

Malondialdehyde was analyzed in plasma using a commercially available assay (Northwest Life Science Specialties, Vancouver, WA) using the method described by Jentzsch et al. [48] The intra-assay coefficient of variation (CV) was 4.7%. Xanthine oxidase activity and hydrogen peroxide were measured in plasma using the Amplex Red reagent method as described by the manufacturer (Molecular Probes, Invitrogen Detection Technologies, Eugene, OR; CV = 4.1 and 4.7%, respectively). Assays for glucose, triglycerides, and cholesterol were performed following standard enzymatic procedures as described by the reagent manufacturer (Thermo Electron Clinical Chemistry; CV<5%). LDL cholesterol was calculated using the Friedwald equation as follows: LDL cholesterol = TC - HDL cholesterol - (TAG/5). Antioxidant capacity was measured in serum using the TEAC following procedures outlined by the reagent provider (Sigma Chemical, St. Louis, MO; CV = 3.7%). Nitrate/nitrite was analyzed in plasma using a commercially available assay (Caymen Chemical, Ann Arbor, MI; CV = 6.4%). HbA1c was analyzed in whole blood following procedures outlined by the reagent provider (Diazyme Laboratories, Poway, CA; CV = 7.2%). Insulin was analyzed in serum using an enzyme linked immunosorbent assay (ELISA) following procedures outlined by the reagent provider (Diagnostic Systems Laboratories, Webster, TX; CV = 6.3%). The homeostatis model assessment (HOMA-IR) was used as an index of insulin resistance [49] and calculated as: [fasting glucose (mg·dL^-1^) × fating insulin (μU·mL^-1^)]/405. C-reactive protein was analyzed in serum using an ultra-sensitive ELISA procedure as described by the manufacturer (Diagnostic Systems Laboratories, Webster, TX; CV = 5.8%).

### Dietary Records

Subjects were instructed to maintain their normal diet and to record their food and beverage intake during the seven days preceding each test meal day (pre and post intervention). Research assistants reviewed portion sizes with subjects through the use of food models, and reviewed all entries with subjects immediately upon diet record return. The records were analyzed for total kilocalories, protein, carbohydrate, fat, vitamins C, A, and E (Food Processor SQL, version 9.9, ESHA Research, Salem, OR). Subjects were given precise instructions regarding abstinence of alcohol consumption, as well as the avoidance of strenuous physical activity during the 24 hours immediately prior to the test meal days, as such activity is known to favorably alter glucose and lipid metabolism [50,51] and result in lower oxidative stress [52].

### Statistical Analysis

Area under the curve (AUC) was calculated for each using the trapezoidal method (AUC_G_) as described in detail by Pruessner et al. [53]. All variables were then analyzed using a 2 (condition) × 2 (pre-post intervention) ANOVA. Fasting (pre-meal) blood samples for all variables were also analyzed between conditions, pre and post intervention. Paired contrasts were used to further analyze effects. Effect size calculations were made using Cohen's *d*. All analyses were performed using JMP statistical software (version 4.0.3, SAS Institute, Cary, NC). Statistical significance was set at P = 0.05. Data are presented as mean ± SEM.

## Results

Although 40 subjects were initially enrolled in the study, only 29 subjects were included in the data analysis due to failure to complete the entire eight weeks of the intervention or a request to be dropped from the study. It should be noted that no subject was dropped due to an adverse outcome associated with the ALCA or placebo supplementation. Rather, loss of interest and personal issues unrelated to the study protocol was the chief reason for subject attrition. Of the 29 subjects remaining, compliance to treatment was 93% for ALCA and 91% for placebo, and not different between conditions (p > 0.05).

**Figure 1:**
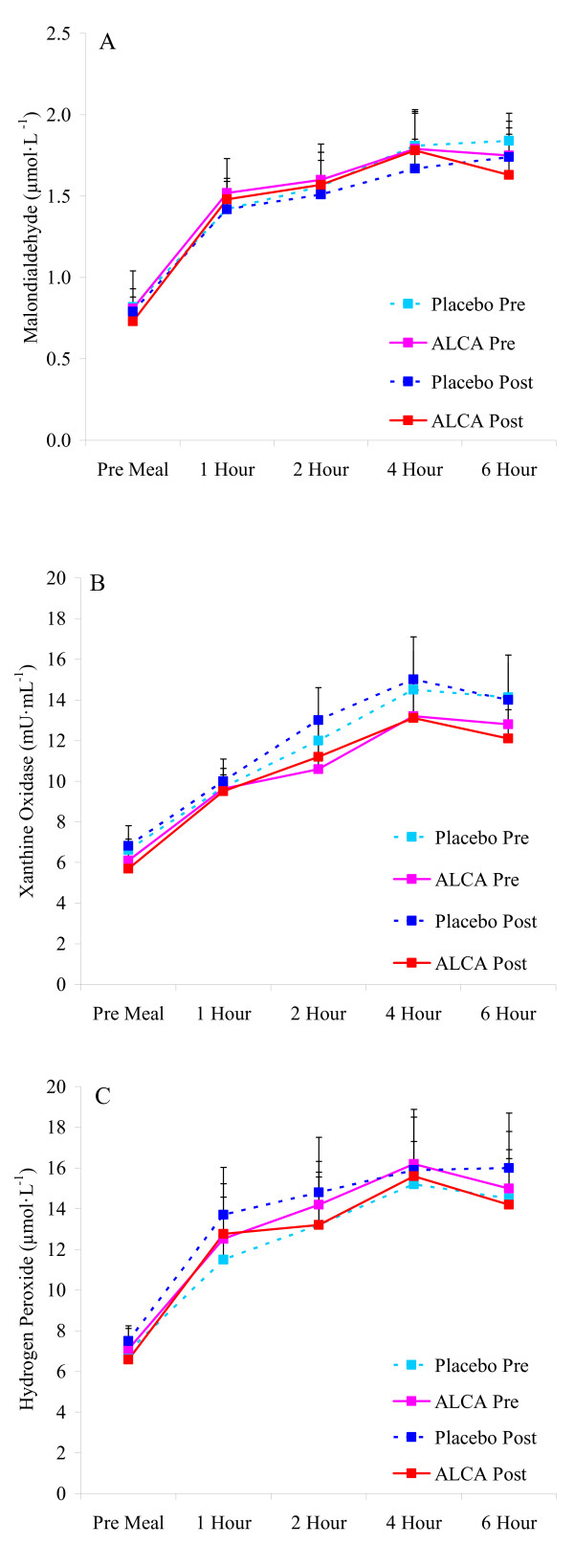
**Blood malondialdehyde (A), xanthine oxidaseactivity (B), and hydrogen peroxide (C) in pre-diabetic subjects before and after an eight week intervention of ALCA or placebo**. Data are mean ± SEM.

**Figure 2:**
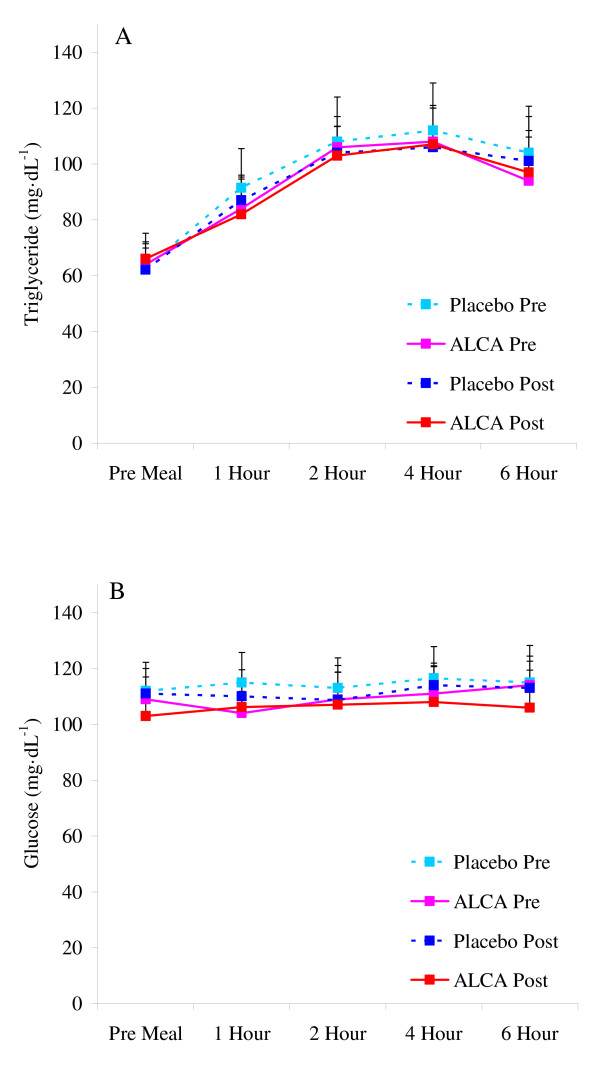
**Blood triglycerides (A) and glucose (B) in pre-diabetic subjects before and after an eight week intervention of ALCA or placebo**. Data are mean ± SEM.

**Figure 3:**
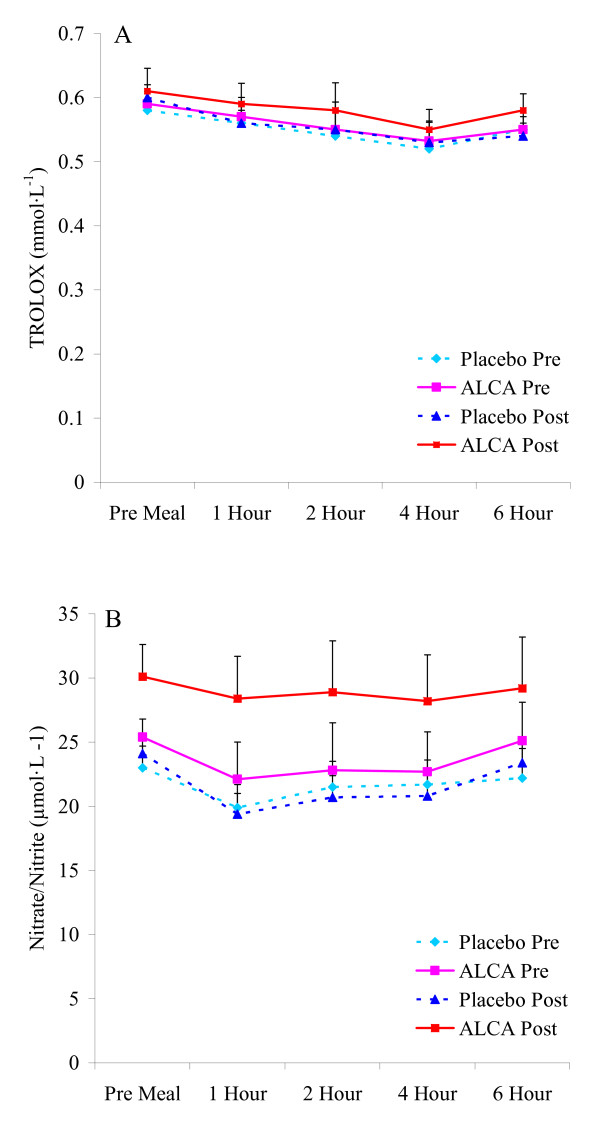
**Blood TEAC (A) and nitrate/nitrite (B) in pre-diabetic subjects before and after an eight week intervention of ALCA or placebo**. Data are mean ± SEM.

**Figure 4:**
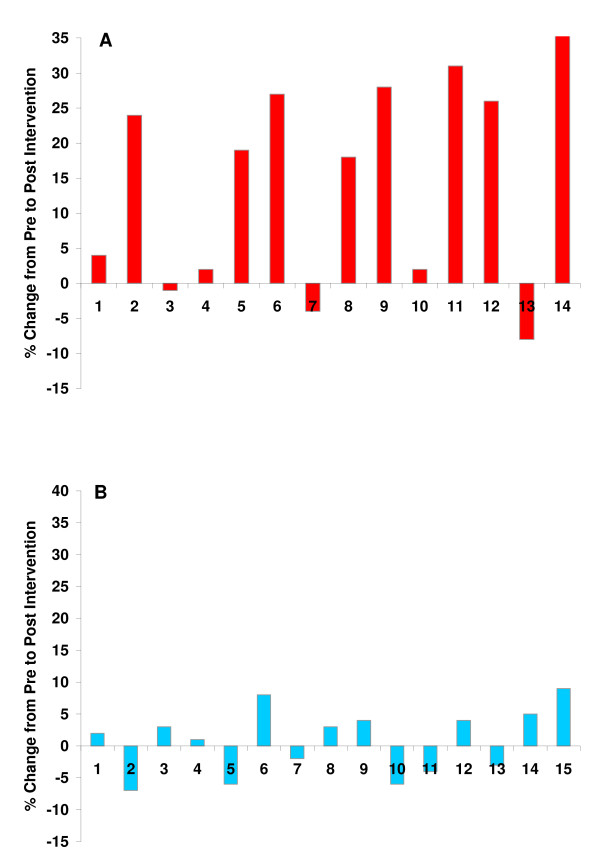
**Individual percent change data from pre to post intervention in fasting nitrate/nitrite for ALCA (A) and placebo (B)**.

No differences were noted between conditions or from pre to post intervention in anthropometric variables, resting heart rate or blood pressure, or fasting metabolic blood parameters (Table 1; p > 0.05; Figures 1, 2, 3). The only exception was higher fasting nitrate/nitrite noted for ALCA compared to placebo post intervention (30.1 ± 2.8 vs. 23.6 ± 2.1 μmol·L^-1^; p = 0.01). This was influenced by the response of a portion of those subjects receiving ALCA, as only eight of the fourteen subjects showed an increase (greater than 5%) in fasting nitrate/nitrite values from pre to post intervention (Figure 4). No differences were noted regarding intake of total kilocalories, total grams of protein, carbohydrate, fat, vitamin C intake, vitamin E intake, or vitamin A intake (Table 2; p > 0.05). No statistically significant differences (p > 0.05) were noted between conditions or pre-post intervention in the AUC for malondialdehyde (Figure 1A), xanthine oxidase activity (Figure 1B), hydrogen peroxide (Figure 1C), triglycerides (Figure 2A), glucose (Figure 2B), TEAC (Figure 3A) or nitrate/nitrite (Figure 3B). Individual subject data for percent change from pre to post intervention for fasting nitrate/nitrite values are presented in Figure 4. All effect size calculations were trivial (*r *< 0.1) except for nitrate/nitrite from pre to post intervention for ALCA, which was considered moderate (*r *= 0.35). AUC data for all variables are shown in Table 3.

**Table 1:** Descriptive characteristics and bloodborne data of pre-diabetic subjects before and after an eight week intervention of ALCA or placebo

Variable	Pre Intervention ALCA	Post Intervention ALCA	Pre Intervention Placebo	Post Intervention Placebo
Age (yrs)	31 ± 3	31 ± 3	35 ± 3	35 ± 3
Height (cm)	172 ± 3	172 ± 3	169 ± 3	169 ± 3
Weight (kg)	85 ± 4	83 ± 5	91 ± 5	90 ± 4
BMI (kg·m^-2^)	28.5 ± 1.9	27.8 ± 1.7	31.7 ± 2.1	32.6 ± 2.4
Body Fat (%)	28 ± 2	26 ± 3	28 ± 2	27 ± 2
Waist (cm)	94 ± 4	92 ± 4	101 ± 5	100 ± 5
Hip (cm)	109 ± 4	106 ± 4	113 ± 5	111 ± 6
Waist:Hip	0.87 ± 0.05	0.87 ± 0.03	0.89 ± 0.04	0.90 ± 0.05
Resting HR (bpm)	73 ± 2	71 ± 2	73 ± 2	72 ± 3
Resting SBP (mmHg)	123 ± 1	121 ± 1	119 ± 1	118 ± 2
Resting DBP (mmHg)	79 ± 1	78 ± 1	80 ± 1	80 ± 1
Glucose (mg·dL^-1^)	109 ± 5	103 ± 5	112 ± 6	111 ± 5
HbA1c (%)	6.6 ± 1.9	6.2 ± 2.2	6.7 ± 1.8	6.6 ± 2.2
Insulin (μU·mL^-1^)	12.1 ± 1.8	11.4 ± 2.1	12.4 ± 1.9	12.7 ± 2.0
HOMA-IR	3.3 ± 1.3	2.9 ± 1.2	3.4 ± 1.4	3.5 ± 1.6
Total Cholesterol (mg·dL^-1^)	195 ± 13	192 ± 14	184 ± 14	179 ± 15
HDL-C (mg·dL^-1^)	51 ± 7	48 ± 6	47 ± 4	50 ± 5
LDL-C (mg·dL^-1^)	127 ± 13	132 ± 11	123 ± 8	117 ± 10
CRP (ng·mL^-1^)	1432 ± 342	1389 ± 328	1652 ± 408	1738 ± 425

Data are mean ± SEM.

No statistically significant differences noted (p > 0.05).

**Table 2:** Dietary data of pre-diabetic subjects during 7 days preceding a test meal

Variable	Pre Intervention ALCA	Post Intervention ALCA	Pre Intervention Placebo	Post Intervention Placebo
Kcal	2425 ± 285	2274 ± 158	2315 ± 217	2380 ± 194
Protein (g)	88 ± 13	84 ± 7	79 ± 8	80 ± 9
CHO (g)	310 ± 33	292 ± 21	275 ± 28	289 ± 26
Fat (g)	93 ± 14	85 ± 9	99 ± 11	100 ± 102
Vitamin C (mg)	102 ± 12	91 ± 16	87 ± 18	76 ± 19
Vitamin E (mg)	10 ± 8	12 ± 5	8 ± 7	7 ± 7
Vitamin A (RE)	3981 ± 1048	4240 ± 845	3745 ± 986	4214 ± 896

Data are mean ± SEM.

No statistically significant differences noted (p > 0.05).

**Table 3:** Postprandial area under the curve data of pre-diabetic subjects before and after an eight week intervention of ALCA or placebo

Variable	Pre Intervention ALCA	Post Intervention ALCA	Pre Intervention Placebo	Post Intervention Placebo
Malondialdehyde (μmol·L^-1^·6 hr^-1^)	9.66 ± 1.33	9.39 ± 1.17	9.56 ± 1.15	9.26 ± 1.07
Xanthine Oxidase (mU·mL^-1^·6 hr^-1^)	67.77 ± 7.70	67.45 ± 9.24	74.11 ± 8.58	76.91 ± 10.39
Hydrogen Peroxide (μmol·L^-1^·6 hr^-1^)	84.76 ± 13.15	81.25 ± 14.87	79.65 ± 12.47	86.45 ± 15.43
Triglyceride (mg·dL^-1^·6 hr^-1^)	585.00 ± 74.22	580.50 ± 73.84	612.95 ± 92.98	597.08 ± 70.43
Glucose (mg·dL^-1^·6 hr^-1^)	658.00 ± 56.27	640.18 ± 73.71	683.00 ± 67.82	679.45 ± 54.46
TEAC (mmol·L^-1^·6 hr^-1^)	3.30 ± 0.19	3.45 ± 0.18	3.25 ± 0.21	3.28 ± 0.19
Nitrate/Nitrite (μmol·L^-1^·6 hr^-1^)	139.50 ± 18.35	172.40 ± 21.75	128.70 ± 11.75	130.45 ± 10.15

Data are mean ± SEM.

No statistically significant differences noted (p > 0.05).

## Discussion

Findings from this investigation indicate that eight weeks of supplementation with ALCA results in an increase in resting nitrate/nitrite in pre-diabetics, but does not promote significant changes in other metabolic or oxidative stress variables measured at rest or post meal. The increase in fasting nitrate/nitrite was influenced primarily by eight of the fourteen subjects receiving ALCA (Figure 4), highlighting the variability in response to dietary supplementation with L-carnitine in regards to blood nitrate/nitrite, a finding we have previously noted using glycine propionyl-L-carnitine [33]. In reviewing subject characteristics in an attempt to determine reasons for the discrepancies in subject response, we noted no apparent differences between "responders" and "non-responders" that would lead us to suggest an association, with the possible exception of slightly lower starting values for some, but certainly not all responders. In this case, it is possible that individuals with lower nitrate/nitrite values may respond to a greater extent to ALCA treatment. Future work is needed to more fully elucidate the reasons for such discrepancies in individual response to ALCA treatment in regards to fasting nitrate/nitrite levels. It is possible that a portion of the increase in blood nitrate/nitrite could have been due to an increase in physical activity of certain subjects assigned to ALCA, as an increase in resting nitrate/nitrite has been reported previously for individuals involved in exercise training [54-56].

Based on the collective data we must reject our hypothesis that ALCA would lead to favorable effects on postprandial oxidative stress and associated biomarkers. However, our findings of a moderate effect size for increase in the AUC for nitrate/nitrite from pre to post intervention with ALCA may have clinical relevance, despite the lack of statistical significance. Of course, this hypothesis needs further investigation, and needs replication in a larger sample of human subjects; in particular in regards to identifying those individuals who are responders to treatment and those who are not.

Unlike the recent work of Volek and coworkers [29] that included only one measure of oxidative stress (malondialdehyde), we included a full panel of commonly assessed oxidative stress biomarkers within the present design. Our failure to note any significant effect of ALCA in regards to this panel provides convincing evidence that no treatment effect related to postprandial oxidative stress is noted with supplementation, at least at the dosage (3 g·day^-1^) and for the duration (8 weeks) provided. This, despite the fact that our chosen treatment included L-arginine, a known precursor to nitric oxide biosynthesis [34], as one component of the supplement. Whereas, the study by Volek and colleagues used L-carnitine, L-tartrate as the supplement of choice [29].

Our data related to nitrate/nitrite provide partial support for the findings of improved FMD with carnitine intake [29], as nitric oxide is known to facilitate vasodilatation by acting on vascular smooth muscle cells [30]. Such an effect may be associated with favorable clinical outcomes, as endothelial dysfunction is linked to cardiovascular morbidity and mortality [8]. However, it is unknown what, if any, clinical relevance both our findings and those of Volek et al. [29] may have. This is particularly true for the extremely small, but statistically significant findings of Volek et al. [29], who noted an increase in postprandial (1.5 hour) FMD from 6.6% to 7.7% with carnitine treatment as compared to a decrease in FMD from 6.6% to 5.8% with placebo. While these findings were noted as statistically significant, their clinical relevance must be questioned, especially considering the somewhat subjective nature of this brachial artery imaging assessment. Moreover, no other differences were noted between carnitine and placebo at the 3.0 and 4.5 hour postprandial measurement times. In fact, FMD was actually higher for placebo at these times compared to carnitine. Clearly, replication of these findings is needed before firm conclusions can be made regarding the impact of supplemental carnitine intake on vascular health and function. This is particularly true considering that no other measures in this study by Volek and colleagues [29] were noted to be significantly effected by carnitine (e.g., triglycerides, malondialdehyde, insulin, interleukin-6, tumor necrosis factor-alpha).

Carnitine may promote increased or maintained nitric oxide in two distinct manners. First, eNOS gene expression has been demonstrated to be increased within cultured human endothelial cells following carnitine incubation [25]. We have recently reported in two studies using human subjects that oral supplementation with carnitine (glycine propionyl L-carnitine) increased nitrate/nitrate at rest [32] and in response to static exercise [33]. Lofreddo and coworkers [31] have also demonstrated that intravenous injection of propionyl L-carnitine increases nitrate/nitrate in patients with peripheral arterial disease. Second, the antioxidant effects of carnitine can provide protection against nitric oxide destruction via superoxide production [57]. L-carnitine has been shown to have effective hydrogen peroxide and superoxide anion scavenging abilities [58], which may minimize the interaction between nitric oxide and superoxide, ultimately leading to decreased formation of peroxynitrite [57] and maintenance of nitric oxide. Hence, carnitine may lead to both the production of nitric oxide, as well as the maintenance of existing nitric oxide.

Our hypothesis for attenuation in oxidative stress biomarkers with ALCA treatment was based on a sound body of literature demonstrating antioxidant benefits of carnitine in both humans [32,59,60] and animals [27,61,62]. Our failure to note significant antioxidant effects related to our chosen biomarkers could be explained in at least two manners.

First, it is possible that the dosage of ALCA used in the present study, in conjunction with the relatively large nutrient load, was not adequate to provide protection against the massive oxidative insult incurred by the test meal. Most previous studies have simply measured oxidative stress biomarkers in a rested, fasted state following carnitine administration. Our use of the test meal may have overwhelmed the antioxidant ability of the ALCA, leading to our null results. This is particularly true considering that our test meal, although not of uncommon size and composition for meals consumed by many overweight/obese individuals, was relatively large compared to some previous studies [63-65]. This may have lead to increased radical production, possibly minimizing any positive effects of the ALCA treatment.

Second, our sample consisted of pre-diabetics, thus deficiencies in postprandial glucose and lipid metabolism likely occurred in response to the test meal. Surprisingly, however, we noted only a minimal blood glucose response to feeding. This is likely due to both the timing of our measurements (i.e., first measurement occurring one hour post feeding), as well as the inclusion of a high amount of dietary fat to the test meal. Almost identical findings have been reported recently by Blendea and colleagues [66]. In addition to being pre-diabetic, our subjects were overweight or obese. We have recently noted that obese individuals experience an exaggerated lipemic and oxidative stress response to high fat, high carbohydrate feeding, as compared to normal weight subjects [45]. This increase in blood triglyceride following feeding is directly linked to superoxide production [67,68], likely mediated by hypertriglyceridemic-induced neutrophilia [69]. Circulating triglycerides may also result in accelerated superoxide production from the abundance of substrate processing and subsequent production of reducing molecules (e.g., NADH, FADH_2_) generated with our large meal [70]. Obese individuals also have lower plasma antioxidants and higher tissue lipid levels which can lead to an increased susceptibility to oxidative stress [71]. Hence, our sample of pre-diabetics may have experienced too great an increase in radical production following the meal, further hampering the ability of the ALCA to provide measurable antioxidant effects. Future work may consider the use of 1) a higher dosage of ALCA, 2) smaller test meals, and 3) the use of metabolically normal test subjects in order to further determine the potential antioxidant benefits of ALCA in relation to postprandial oxidative stress and associated biomarkers.

## Conclusion

Eight weeks of supplementation with ArginoCarn^® ^results in an increase in resting nitrate/nitrite in pre-diabetic subjects, which may allow for higher overall circulating nitrate/nitrite following intake of a high kilocalorie, high fat and carbohydrate meal. This ingredient also results in a slight improvement in glucose metabolism, as evidenced by minor decreases in blood glucose, insulin, and HbA1c (which may have been more pronounced with longer treatment, as HbA1c has a half-life equal to approximately 12 weeks [72]). However, ALCA does not result in significant changes in other metabolic or oxidative stress variables measured at rest or after feeding. It is possible that a greater dosage and/or duration of treatment of ALCA is needed for attenuation of postprandial oxidative stress. Moreover, such factors as the size and composition of the test meal, in addition to abnormal glucose metabolism and excess body weight/fat of our test subjects, may have contributed to the null findings. That is, any potential antioxidant effect of ALCA may have been overwhelmed by the oxidant burden of the test meal, coupled with potentially exacerbated radical generation and decreased antioxidant defense of our test subjects. The increase in nitrate/nitrite may indicate positive effects related to vascular function. Of course, additional study is necessary to confirm this hypothesis.

## Competing interests

Funding for this work was partially provided by Sigma-tau HealthScience. The authors nor the University of Memphis directly endorse the dietary supplement used in this investigation. RJB has been the recipient of research support from the project sponsor since 2005, and is periodically involved in scientific writing for the sponsor.

## Authors' contributions

RJB was responsible for the study design, biochemical work, statistical analyses, and manuscript preparation; KHFW and PST were responsible for data collection, blood collection and processing, and manuscript preparation. All authors read and approved of the final manuscript.
